# Isolated congenital complete heart block in a five-year-old seronegative girl born to a woman seropositive for human immunodeficiency virus: a case report

**DOI:** 10.1186/s13256-016-1082-5

**Published:** 2016-10-19

**Authors:** Pedro Pallangyo, Isaac Mawenya, Paulina Nicholaus, Henry Mayala, Amida Kalombola, Godwin Sharau, Naiz Majani, Mohamed Janabi

**Affiliations:** 1 Department of Cardiovascular Medicine, The Jakaya Kikwete Cardiac Institute, P.O Box 65141, Dar es Salaam, Tanzania; 2 Department of Pediatric and Child Health, Muhimbili National Hospital, P.O Box 65000, Dar es Salaam, Tanzania; 3 Department of Pediatric Cardiology, The Jakaya Kikwete Cardiac Institute, P.O Box 65141, Dar es Salaam, Tanzania

**Keywords:** Congenital complete heart block, Congenital third-degree AV block, Isolated congenital heart block, Recurrent syncopal attacks, Symptomatic bradycardia, Case report

## Abstract

**Background:**

Congenital complete heart block is a life-threatening condition which is highly associated with autoimmune and connective tissue disorders. Presence of maternal autoantibodies for associated conditions increases the risk of delivering a child with congenital complete heart block, however, less than a half of all women with such antibodies are symptomatic even after delivery. Mortality rate is highest during the neonatal period (45 %) and about two-thirds of all cases will require permanent pacing at some point in their lives.

**Case presentation:**

We report a case of isolated complete heart block in a 5-year-old HIV-free girl of African descent born to an HIV-infected woman with no prior history of autoimmune disorders. She was referred to us with chief complaints of recurrent syncopal attacks and effort intolerance since birth. A physical examination was unremarkable except for her being small for her age (body mass index 16.3 kg/m^2^) and bradycardia. Her vital signs were within acceptable range with the exception of her pulse rate, which ranged between 22 and 34 beats/minute. An echocardiogram revealed a sinus bradycardia, otherwise a structurally normal heart. An electrocardiogram showed atrioventricular dissociation in keeping with third-degree atrioventricular block. The child underwent a permanent epicardial pacemaker insertion and has been symptom-free following pacing.

**Conclusions:**

Despite its infrequency and life-threatening potential, patients with congenital complete heart block have an excellent survival rate with timely diagnosis and intervention. An incidental detection of bradycardia in a fetus during routine obstetrical ultrasound examination should increase the index of suspicion for congenital complete heart block and warrant a screening for associated maternal autoantibodies.

## Background

Congenital complete heart block (CCHB) is a rare, permanent, irreversible and life-threatening anomaly that is present in about 1 of every 20,000 live-born infants [1]. It may be diagnosed in utero, during infanthood or early childhood and is frequently associated with maternal autoantibodies, or rarely with a congenital structural deformity of the heart [2]. The risk of delivering a child with CCHB is higher among women who are anti-SSA/Ro or anti-SSB/La positive and in those with autoimmune hypothyroidism [3]. The transplacental transfer of maternal autoantibodies to SSA/Ro or SSB/La ribonucleoproteins may lead to inflammation and ultimately fibrosis of the fetal conduction system [4]. Interestingly, over 50 % of mothers carrying such autoantibodies are asymptomatic and are identified only after they have children with a heart block [4]. Furthermore, the risk of CCHB recurrence among women with associated autoantibodies rises to 17 % in the second offspring and may rise up to 50 % in subsequent deliveries [4]. For instance, infants with neonatal lupus erythromatosus (NLE) have up to a 30 % risk of developing CCHB compared to infants without it and such infants comprise over 90 % of all CCHB cases [5, 6].

Clinically, CCHB presents with recurrent fainting attacks, a slow pulse rate and electrocardiographic (ECG) findings in keeping with complete heart block [1, 2, 4]. About two-thirds of all CCHB cases will require permanent pacing, which provides an excellent prognosis especially among those with the isolated form [7]. Overall mortality rate in CCHB is about 20 %, it is higher in utero (27 %) or during early infancy (45 %) and is associated with delayed pacing therapy [7, 8]. Comparatively, CCHB cases with comorbid cardiac structural malformations have the worst prognosis [2]. We report a case of isolated complete heart block in a 5-year-old girl with a history of recurrent syncopal attacks and effort intolerance since birth.

## Case presentation

A 5-year-old HIV-free girl of African descent was referred to us with chief complaints of recurrent syncopal attacks and effort intolerance since birth. She is a third-born child to a 38-year-old, single, HIV-infected woman. Her mother first gave birth at the age of 27, she was HIV-free then but the baby was born with spina bifida and anencephaly and survived for just a few hours after birth. She then gave birth to a baby girl 2 years later; the child is healthy and alive until today. She became pregnant again 4 years after the birth of her second child. During an antenatal clinic visit for this pregnancy, she tested negative for hepatitis B and her syphilis screening was nonreactive. She was never screened for rubella or autoimmune conditions. She had low hemoglobin concentration throughout (9.4–10.9 g/dL), but received hematenics, antihelminthics, antimalarial and tetanus toxoid immunization as per protocol. She was diagnosed with HIV infection during the antenatal clinic visit at 24 weeks’ gestation and was started on antiretroviral medication. She reported that she took the medication for 3 days only and decided to stop because she did not want her family to know of her HIV serostatus. She had severe hyperemesis gravidarum during her first trimester and was hospitalized seven times due to this. Her pregnancy thereafter was uneventful and she gave birth to a full-term baby girl weighing 2.3 kilograms who scored 7/10 on the APGAR scale. The child was given nevirapine at birth and she was instructed to continue giving it for 6 weeks, but for the same reasons she stopped her child’s nevirapine on the third day. She exclusively breastfed her child for 6 months and never resumed her or her child’s antiretroviral medication during this period. The mother denied any symptoms suggestive of hypothyroidism, connective tissue or autoimmune disorders before, during, and after pregnancy.

During her third child’s first few weeks of life, the mother observed that she was breastfeeding poorly and was sleeping for longer duration compared to her sister at her age. At about 8 weeks, it was observed that the child would go into an unarousable state on exertion or while breastfeeding. This state lasted between 30 minutes and 1 hour, and upon regaining consciousness, the child was lethargic. The child never presented with a crying episode or convulsions prior to her loss of consciousness. The child was taken to a health facility and the mother was told that her child’s heart was beating slower than usual but was assured that it would go away with time. She continued to present with syncopal attacks until the age of 2 years but was never admitted for this or any other illness and had normal developmental milestones. She was free of such episodes for about 3 years, however, she was mostly lethargic with a decreased effort capacity, had a poor weight gain and was generally inactive for her age. Three weeks prior to this index admission, she presented again with episodes of loss of consciousness which persisted until she came to our attention. She had experienced between three and five episodes per day, each lasting for about an hour and after regaining consciousness she slept for up to 12 hours. This time round, minor activities including swallowing and rising from a lying position led to syncope. Since birth, the child had never had features of heart failure or a transient rash suggestive of neonatal lupus erythromatosus. Furthermore, the mother denies any prescription of steroids for herself or her child between pregnancy and now.

**Fig. 1:**
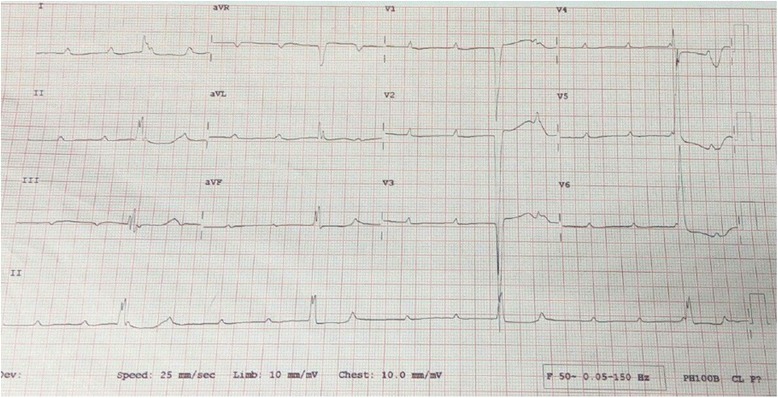
Electrocardiogram showing atrioventricular dissociation in keeping with the diagnosis of complete heart block

Upon admission to our institute, the child underwent a thorough physical examination and a series of investigations. The physical examination was unremarkable except for her being small for her age (body mass index (BMI) 16.3 kg/m^2^) and bradycardia. Her vital signs were within acceptable range with the exception of her pulse rate, which ranged between 22 and 34 beats/minute. She had normal electrolytes, renal and liver function, full blood count and random blood sugar test results. Serology results for HIV and hepatitis (B and C) were negative. An echocardiogram revealed a sinus bradycardia, otherwise a structurally normal heart. An ECG showed AV dissociation in keeping with third-degree atrioventricular (AV) block, Fig. 1. Anti-SSA/Ro and anti-SSB/La antibodies tests are unavailable in the country and were not screened for, however, the mother tested negative for anti-double-stranded deoxyribonucleic acid (dsDNA), antinuclear antibodies (ANA) and antineutrophil cytoplasmic antibodies (ANCA) autoantibodies. The child underwent a permanent epicardial pacemaker insertion and has been symptom-free following pacing.

## Discussion

Complete congenital heart block is a potentially fatal condition although in this modern era the prognosis is excellent. In the developed world, the majority of patients are diagnosed in utero, however, in resource-limited settings where fetal echocardiography is a rare practice, diagnosis is exclusively made during infancy [9]. Early diagnosis of isolated CCHB, which is highly associated with autoimmune disorders, remains a challenge even in the best centers because the majority of women are asymptomatic even after delivery [1, 4, 5, 8]. When CCHB is diagnosed in utero in a woman with autoantibodies, several therapeutic approaches including use of steroids, sympathomimetics, plasmapheresis, and fetal pacing have been implicated [10]. Children with CCHB exhibiting symptoms early in life are regarded high risk and necessitate urgent pacing. A pacemaker aims to prevent sudden cardiac death, provide symptomatic relief, and improve the quality of life [2].

In the case presented, the child was symptomatic from birth but a definitive diagnosis of CCHB was made at around the age of 5. While mortality rate due to CCHB is reportedly high during the neonatal period, our case is very fortunate to have survived until age 5 without any intervention despite the presence of recurrent syncopal attacks since birth. This case, despite undergoing all the diagnostic hurdles, served as a learning platform to practitioners in our center as this was the first-ever case of CCHB to be diagnosed, paced, and documented in Tanzania.

## Conclusions

In conclusion, despite of its infrequency and life-threatening potential, patients with CCHB have an excellent survival rate with timely diagnosis and intervention. Furthermore, among women with established associated autoimmune conditions, frequent echocardiographic fetal heart rate assessment and necessary in utero interventions are essential for better fetal outcomes. On the other hand, incidental detection of bradycardia in a fetus during routine obstetrical ultrasound examination should increase an index of suspicion for CCHB and warrant a screening for associated maternal autoantibodies.

## Abbreviations

ANA: antinuclear antibodies
ANCA: antineutrophil cytoplasmic antibodies
AV: atrioventricular
CCHB: complete congenital heart block
dsDNA: double-stranded deoxyribonucleic acid
ECG: electrocardiogram
NLE: neonatal lupus erythromatosus
